# Study of the Synthesis of Multi-Cationic Sm-Co-O, Sm-Ni-O, Al-Co-O, Al-Ni-O, and Al-Co-Ni-O Aerogels and Their Catalytic Activity in the Dry Reforming of Methane

**DOI:** 10.3390/gels10050328

**Published:** 2024-05-11

**Authors:** Jaroslav Cihlar, Serhii Tkachenko, Vendula Bednarikova, Jaroslav Cihlar, Klara Castkova, Martin Trunec, Ladislav Celko

**Affiliations:** 1 Central European Institute of Technology, Brno University of Technology, Purkyňova 123, 612 00 Brno, Czech Republic; serhii.tkachenko@ceitec.vutbr.cz (S.T.); vendula.bednarikova@ceitec.vutbr.cz (V.B.); klara.castkova@ceitec.vutbr.cz (K.C.); martin.trunec@ceitec.vutbr.cz (M.T.); ladislav.celko@ceitec.vutbr.cz (L.C.); 2 Institute of Materials Science and Engineering, Brno University of Technology, Technicka 2, 616 69 Brno, Czech Republic; 3 Institute of Rock Structure and Mechanics of the Czech Academy of Sciences, V Holešovičkách 94/41, 182 09 Praha, Czech Republic; cihlar@irsm.cas.cz

**Keywords:** multi-cationic aerogels, dry reformation of methane, cobalt, nickel, nanoparticles, carbon deposition

## Abstract

Dense multi-cationic Sm-Co-O, Sm-Ni-O, Al-Co-O, Al-Ni-O, and Al-Ni-Co-O oxide aerogels were prepared by epoxide-driven sol–gel synthesis. Catalysts for dry reformation of methane, Sm_2_O_3_/Co, Sm_2_O_3_/Ni, Al_2_O_3_/Co, Al_2_O_3_/Ni, Al_2_O_3_/Co, and Ni were prepared by reduction of aerogels with hydrogen and their catalytic activities and C-deposition during dry reformation of methane were tested. Catalytic tests showed high methane conversion (93–98%) and C-deposition (0.01–4.35 mg C/g_cat_**^.^**h). The highest content of C-deposits after catalytic tests was determined for Al_2_O_3_/Co and Al_2_O_3_/Ni catalysts, which was related to the formation of Al alloys with Co and Ni. A uniform distribution of Co^0^ and Ni^0^ nanoparticles (in the form of a CoNi alloy) was found only for the Al_2_O_3_/Co and Ni catalysts, which showed the highest activity as well as low C deposition.

## 1. Introduction

Climate change is mainly related to burning fossil fuels that produce carbon dioxide. Another greenhouse gas is methane, which comes mainly from fossil sources. One of the essential ways of reducing emissions of these greenhouse gases, CO_2_ and CH_4_, is dry reformation of methane (DRM), whose important product is hydrogen or synthesis gas. Due to its ecological and economic benefits, many professional reviews and scientific works are devoted to the dry reformation of methane [1]. These works aim to bring new catalytic systems with high catalytic activity at the lowest possible temperature, which are long-term stable, have low C-deposition, and are affordable [2]. These requirements are currently best met by heterogeneous catalytic systems containing catalytically active particles of transition metals, especially Ni and Co, deposited on the surfaces of inorganic oxides [3,4]. Their catalytic efficiency depends on the interplay of acid–base and redox properties of metal particles and oxide substrates during the transfer of electrons and oxide ions between the reactants participating in DRM (CH_4_, CO_2_, H_2_, and CO). The structural properties of reforming catalysts are no less critical, such as surface size, particle size, the composition of active centers, and their localization on substrate surfaces [5]. The chemical and phase composition, architecture, and surface morphology of reforming catalysts depend on their preparation technology. Among the most valuable are the sol–gel methods, which allow control of the homogeneity of the reactants and the dispersions that arise from them. It is generally considered that the homogeneity of dispersions is one of the prerequisites for forming homogeneously distributed active centers on the surface of heterogeneous catalysts [6]. Aerogels are three-dimensional porous materials with interconnected micropores, often prepared by sol–gel methods and supercritical gel drying, which can preserve the large surface texture of the raw gel [7]. Inorganic oxide aerogels containing several cations can be transformed by hydrogen reduction into heterogeneous catalysts composed of an oxide substrate and an active metal. Achieving the transition of a sol (dispersion) to a polymer gel in multi-cationic systems and creating a sufficiently homogeneous distribution of cations in the gel is not easy and depends primarily on the course of hydrolysis and condensation of reactants during gelation [8].

The work is focused on the study of di-cationic aerogels composed of two oxides, one of which is “irreducible” with hydrogen and serves as a substrate and the other is “reducible”, which is excreted on the surface of the substrate through reduction and forms active centers for dry reformation of methane. The work’s primary goal is to prepare di-cationic aerogels, where both cations are “homogeneously” dispersed in the aerogel structure. During reduction, the reducible cation diffuses through the aerogel structure and is reduced by hydrogen to an active metal at the surface of the substrate. The composition of di-cationic aerogels is designed so that, by reduction, catalytic systems containing oxide substrates (Sm_2_O_3_ and Al_2_O_3_) with different interactions with the most catalytically active general transition elements (Co and Ni) are created. The three-cationic aerogel Al-Ni-Co-O was included in the work in order to verify the higher activity and lower C-deposition compared to the di-cationic Al-Co-O and Al-Ni-O catalysts. The work contains the study results of epoxide-driven synthesis of dense oxide aerogels Sm-Co-O, Sm-Ni-O, Al-Co-O, Al-Ni-O, and Al-Ni-Co-O. The work also includes the results of the study of the reduction in aerogels with hydrogen to reforming catalysts, Sm_2_O_3_/Co, Sm_2_O_3_/Ni, Al_2_O_3_/Co, Al_2_O_3_/Ni, Al_2_O_3_/Co, Ni and testing of their catalytic activity and C deposition during dry methane reforming. We see the benefit of the work in a new perspective on C-deposition in aerogel Al_2_O_3_/Co and Al_2_O_3_/Ni catalysts.

## 2. Results and Discussion

### 2.1. Synthesis and Characterization of Aerogels

Sm, Al, Co, and Ni chlorides with six crystalline water molecules were used to synthesize aerogels. Chlorides with the mentioned cations had an octahedral configuration in which water molecules are bound in the inner-sphere of the aqua complex and chloride ions lie in the outer-sphere of the aqua complex (x = 2, 3) [M(H_2_O)_6_]^x+^· xCl^−^ [8,9,10,11,12].

All cations used have a high charge/radius ratio (charge density) and their hydrated cations (aqua cationic complex) in aqueous-alcoholic solutions were subject to hydrolysis according to Equation (1) [13], as follows:

[M(H_2_O)_6_]^r+^ + H_2_O = [M(OH)(H_2_O)_5_]^(r−1)+^ + H_3_O^+^(5) (M = Co^2+^, Ni^2+^, Al^3+^, Sm^3+^) (1)

The greater the charge of the cation and the smaller the size of the ion, the more easily the ion polarizes coordinated water molecules. This weakens the OH bond in the water molecule and releases the H^+^ (or H_3_O^+^) ion into the solution. The charge density of cations (C/mm^3^) increases in the order Sm(86) < Ni(134) < Co(155) < Al(364) [14,15]. The hydrolysis and acidity of chloride solutions of these cations increase in the same order. By pumping out protons (H_3_O^+^) from the hydrolyzed aqua cationic complexes, olation and the formation of μ-hydroxo-bridged complexes first occur according to Equation (2), as follows:

2[M(H_2_O)_5_(OH)]^(r−1)+^ + H^+^ = [(H_2_O)_5_M-OH-M(H_2_O)_5_] ^(2r−1)+^ + H_2_O (2)

By pumping out protons from μ-hydroxo-bridged metal complexes, oxolation and the formation of μ-oxo-bridged metal complexes occurs according to Equation (3), as follows:

[(H_2_O)_5_M-OH-M(H_2_O)_5_]^(2r−1)+^ = [(H_2_O)_5_M-O-M(H_2_O)_5_]^(2r−2)+^ + H^+^ (3)

Slow hydrolysis (deprotonation), olation, and oxolation (condensation) lead to the formation of polymeric species in gels. In the epoxide-driven method, the epoxide (propylene oxide) acts as a base, slowly reacting by the epoxide ring-opening mechanism with protons released by hydrolysis and oxolation, thus promoting the creation of gels according to Equation (4) [16,17], as follows:
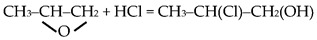
(4)

In multication systems, namely Sm-Co-O, Sm-Ni-O, Al-Co-O, Al-Ni-O, and Al-Ni-Co-O as well as not only Sm-O-Co, Sm-O-Ni, Al-O-Co, Al-O-Ni-Co bonds but also “homo-metallic” M_i_-O-M_i_ bonds, can be formed by olation and oxolation. Different rates of hydrolysis and condensation of individual aqua-cationic species can lead to inhomogeneities in gels and phase separation in multimetallic aerogels [17].

**Figure 1:**
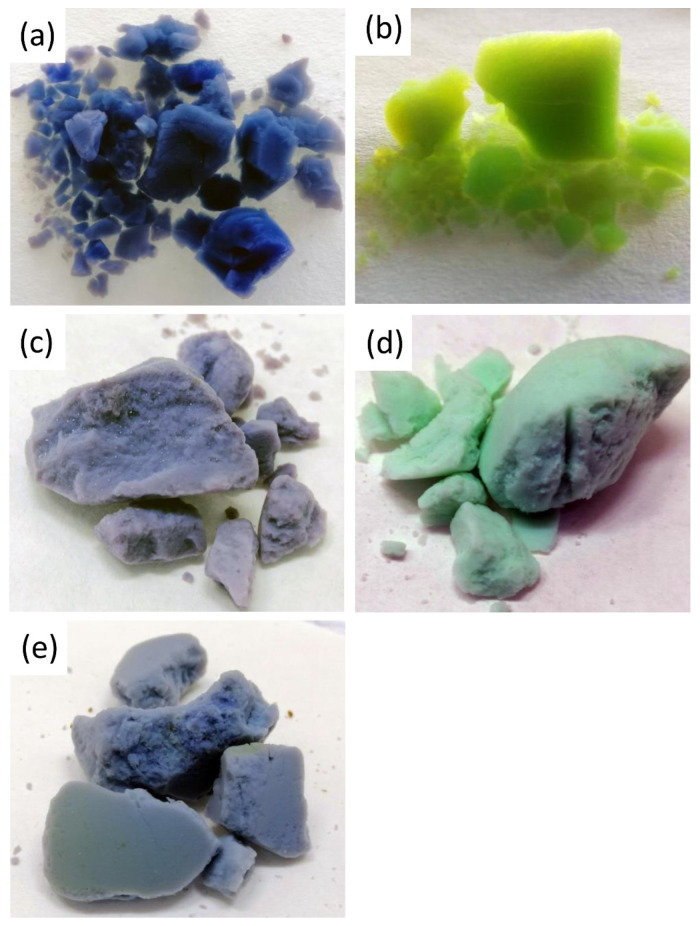
Images of supercritically dried aerogels: (**a**) Sm-Co-O, (**b**) Sm-Ni-O, (**c**) Al-Co-O, (**d**) Al-Ni-O, and (**e**) Al-Co-Ni-O.

The precursor chlorides of Sm, Al, Co, and Ni formed in epoxy-driven synthesis of dense and visually homogeneous aerogels (Figure 1).

Hydrolysis and condensation rates of precursors of aerogels were manifested on values gelation times (t_gel_) listed in Table 1. The longest t_gel_ was observed at gelation di-cationic gels containing Sm^3+^ cations; the shortest times were found for gels containing Al^3+^ cations. These findings are consistent with the charge density values of Sm^3+^ and Al^3+^ cations (Table 1). If the values of t_gel_ given in Table 1 are compared with the published values for SmCl_3_ (12 min, [18]) and AlCl_3_ (3 min, [19]), it can be seen that the gelation times of gels were affected primarily by chlorides containing Sm^3+^ and Al^3+^ ions.

**Figure 2:**
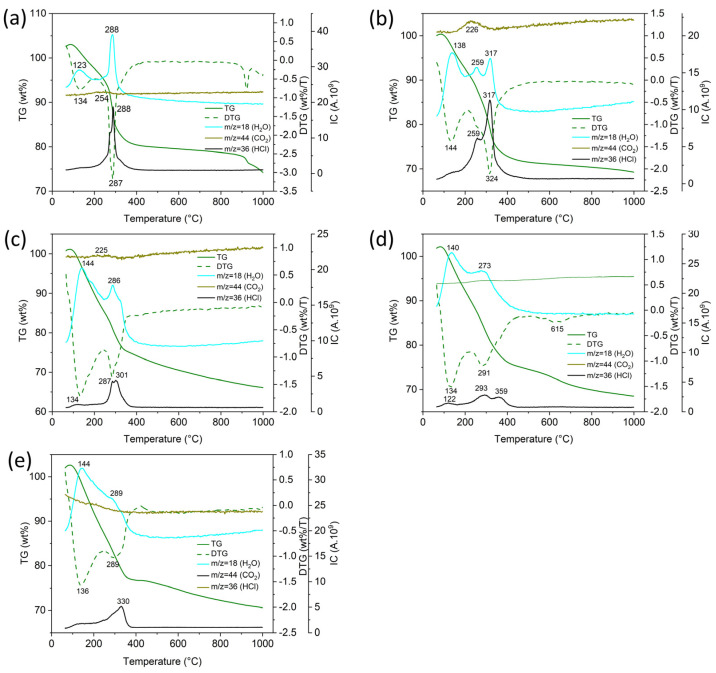
Simultaneous TGA/MS profiles of supercritically dried aerogels: (**a**) Sm-Co-O, (**b**) Sm-Ni-O, (**c**) Al-Co-O, (**d**) Al-Ni-O, and (**e**) Al-Co-Ni-O.

The results of simultaneous TGA/MS analysis of supercritically dried Sm-Al-Co-Ni-O aerogels are shown in Figure 2 and Table 2. In the temperature range of 50–220 °C, the aerogels lost 6–15% of their mass due to the evaporation of physically adsorbed water; see endothermic peaks (Q)_DSC_ = −122 to −254 J/g and peaks *m*/*z* = 18 (H_2_O) and (T_max_)_H2O_ = 123 to 144 °C. At a temperature of 220 to 600 °C, the weight loss of the aerogel was 10 to 22%; see exothermic peaks (Q)_DSC_ = 157 to 642 J/g and peaks *m*/*z* = 18 (T_max_)_H2O_ = 273 to 317 °C. Peaks of CO_2_ (*m*/*z* = 44) were detected in the temperature range 220 to 600 °C associated with the oxidation of organic molecules formed by the reaction of propylene oxide with chloride precursors (see exothermic peaks (Q)_DSC_ and (T_max_)_CO2_ = 288 to 359 °C. In the MS spectra of gaseous products of thermal oxidation of aerogels, traces of Cl were also detected, which were released in the form of HCl (*m*/*z* = 36) from M-Cl groups or chloropropyl alcohol. It follows from the STA/MS analysis that the multi-cationic dried aerogels contained hydroxides-oxides of Sm, Al, Ni, Co, and the content of M-OH groups, which was in the range of 1.2 to 2.5 mol/100g of aerogel, did not depend on the precursors used.

**Figure 3:**
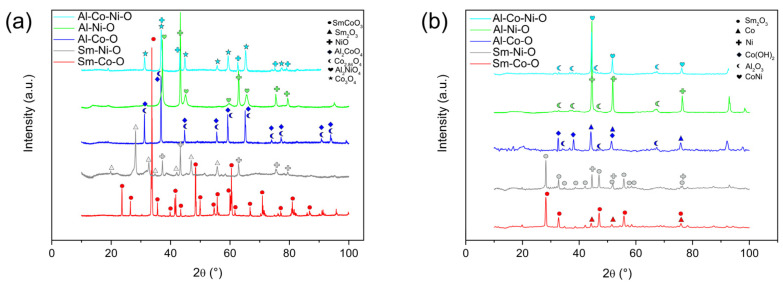
XRD patterns of calcined (**a**) and reduced (**b**) aerogels.

The phase composition of calcined aerogels (T = 900 °C/10 h) can be seen in Figure 3. Except for the single-phase SmCoO_3-δ_ perovskite, calcination resulted in two-phase or three-phase products (Al-Co-Ni-O). Spinel phases appeared in Al-Co-O and Al-Ni-O aerogels. Calcination was performed on uncrushed samples at a relatively short exposure time to verify the homogeneity in the distribution of cations in aerogels prepared by epoxy-driven synthesis. It was assumed that the formation of single-phase products would manifest the homogeneous composition of the dried aerogels. If we neglect the different activation energies of high-temperature reactions of mixed oxides (Sm-Co-O, Sm-Ni-O, Al-Co-O, Al-Ni-O, and Al-Ni-Co-O), the formation of multiphase products indicates an inhomogeneous distribution of cations in aerogels leading to phase separation.

**Figure 4:**
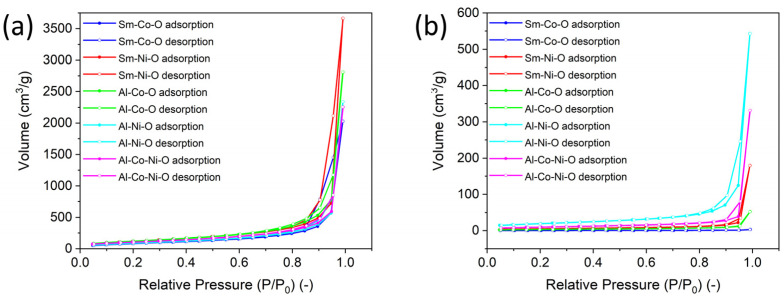
Nitrogen adsorption and desorption isotherms of thermally treated (**a**) and calcined (**b**) aerogels.

The results of BET analysis of the surface of aerogels heat-treated at 500 °C/h and calcined at 900 °C/10 h are shown in Figure 4 and Table 3. Gas adsorption experiments revealed that powders after both thermal treatments exhibited similar features of physisorption behavior. All adsorption/desorption isotherms can be classified as Type II isotherms [20]. Such isotherms are associated with an open and stable external surface of non-porous or macroporous powders. Small hysteresis loops (Type H3) observed on some isotherms (Sm-Co-O, Sm-Ni-O) resulted from a substantial agglomeration of primary particles. The particle agglomerates exhibited macropores that were not filled by the condensed adsorbent. The specific surface area of individual samples is shown in Table 2. Heat-treated aerogels at a relatively low temperature (500 °C/1 h) had a very high surface area of up to 470 m^2^/g. The aerogels did not have a mesoporous structure and were formed by agglomerates of hydroxide-oxide nanoparticles. The surface area decreased substantially after calcination at 900 °C/10 h due to particle coarsening and sintering.

### 2.2. Reduction in Calcined Aerogels

**Figure 5:**
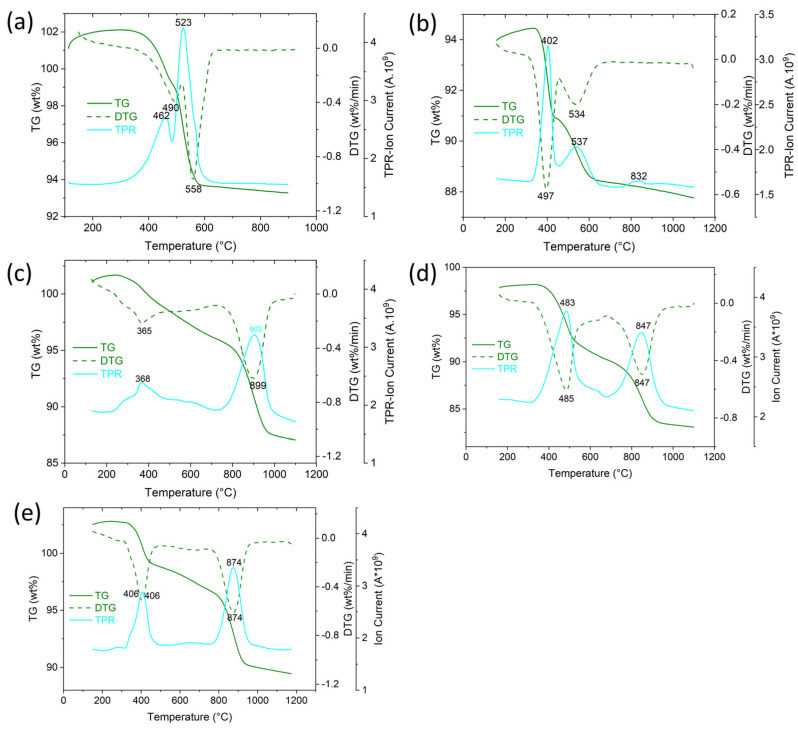
TGA and H_2_-TPR profiles of calcined aerogels: (**a**) Sm-Co-O, (**b**) Sm-Ni-O, (**c**) Al-Co-O, (**d**) Al-Ni-O, and (**e**) Al-Co-Ni-O.

Since the catalytically active sites must contain metal particles (Co^0^, Ni^0^, Ni^0^Co^0^), the calcined aerogels were activated by reduction. Reducibility aerogels were studied by TPR-H_2,_ and the results are shown in Figure 5. TPR-H_2_ aerogel profiles Sm-Co-O contains two reduction peaks, T_max_ = 462 and 523 °C. The first peak corresponds to the reduction Co^3+^ → Co^2+^ and the second peak corresponds to the reduction Co^2+^ → Co^0^ [21,22,23,24]. The TPR-H _2_ profile of the Sm-Ni-O aerogel has two peaks, a low-temperature one (T_max_ = 402 °C), which is related to the reduction in Ni^3+^ to Ni^2+^, and a high-temperature one (T_max_ = 537 °C), which corresponds to the reduction Ni^2+^ to Ni^0^. The reduction behavior of Sm-Ni-O is similar to that of La-Ni-O [25,26,27]. Aerogels containing Al: Al-Co-O, Al-Ni-O and Al-Ni-Co-O differed from aerogels containing Sm by a significantly higher temperature of the second reduction peak. While the first reduction peak, corresponding to the reduction in Co^3+^, Ni^3+^ to Co^2+^ and Ni^2+,^ was similar to the case of Sm aerogels in the range of 368 to 483 °C, the second reduction peak corresponding to the reduction in Co^2+^, Ni^2+^ → Co^0^, Ni^0^ reached values of 847 to 903 °C. The high reduction temperatures of Al-containing aerogels showed that Co and Ni ions were tightly bound in Al-containing phases (spinels Al-Ni-O, Al-Co-O, Al-Co-Ni-O). From this point of view, the binding of Co and Ni ions in the perovskite phases was weaker.

**Figure 6:**
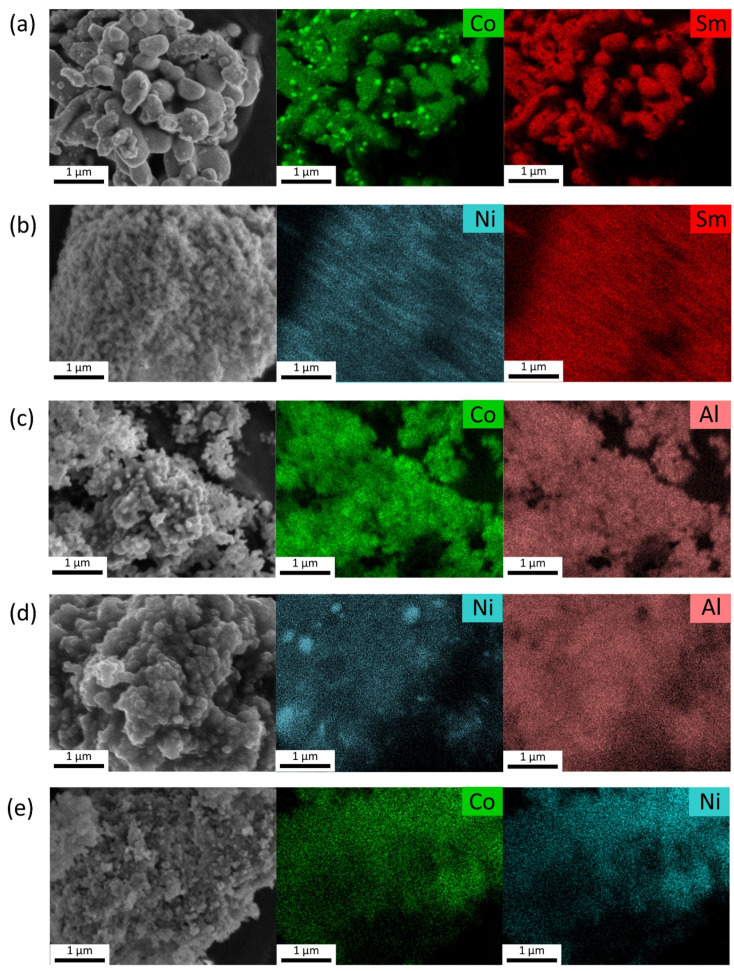
SEM image and EDX elemental mapping of the reduced aerogels: (**a**) Sm-Co-O, (**b**) Sm-Ni-O, (**c**) Al-Co-O, (**d**) Al-Ni-O, and (**e**) Al-Co-Ni-O.

Figure 6 shows the field emission scanning electron microscopy (FESEM) and elemental mapping of reduced aerogels. The images show round particles of the substrates (Sm_2_O_3_, Al_2_O_3_) with a size of several micrometers and micrometer-sized pores formed by sintering during the reduction in aerogels. The distribution of Co^0^ on the surface of Sm-Co-O and Al-Co-O aerogels is significantly heterogeneous. In addition to small, several nanometer-sized nanoparticles uniformly deposited on the surface, spherical particles or clusters of 500–1000 nm size are present. A similar distribution of Ni^0^ is seen on the surface of Al-Ni-O and Sm-Ni-O aerogels. A uniform distribution of Co^0^ and Ni^0^ nanoparticles (probably in the form of a CoNi alloy) without large spherical particles is evident in the Al-Co-Ni-O aerogel. Large particles could form in two-phase (Sm-Ni-O, Al-Co-O, Al-Ni-O) aerogels so that one phase was reduced more easily and quickly (NiO, Co_3_O_4_) than the other (spinel phase). In the case of a single-phase aerogel (Sm-Co-O), the seeds could be formed in the low-temperature phase of the reduction. The practically homogeneous distribution of CoNi nanoparticles on the surface of the substrate is attributed to the interaction of Co^0^ and Ni^0^ nuclei during reduction and their growth at a constant rate without forming agglomerates. Elemental compositions of the reduced aerogels were qualitatively investigated using SEM-EDX and the results are listed in Table 4. These results confirm that aerogels comprise their major chemical elements and do not contain any other elements.

### 2.3. Catalytic Performance of Reduced Aerogels

**Figure 7:**
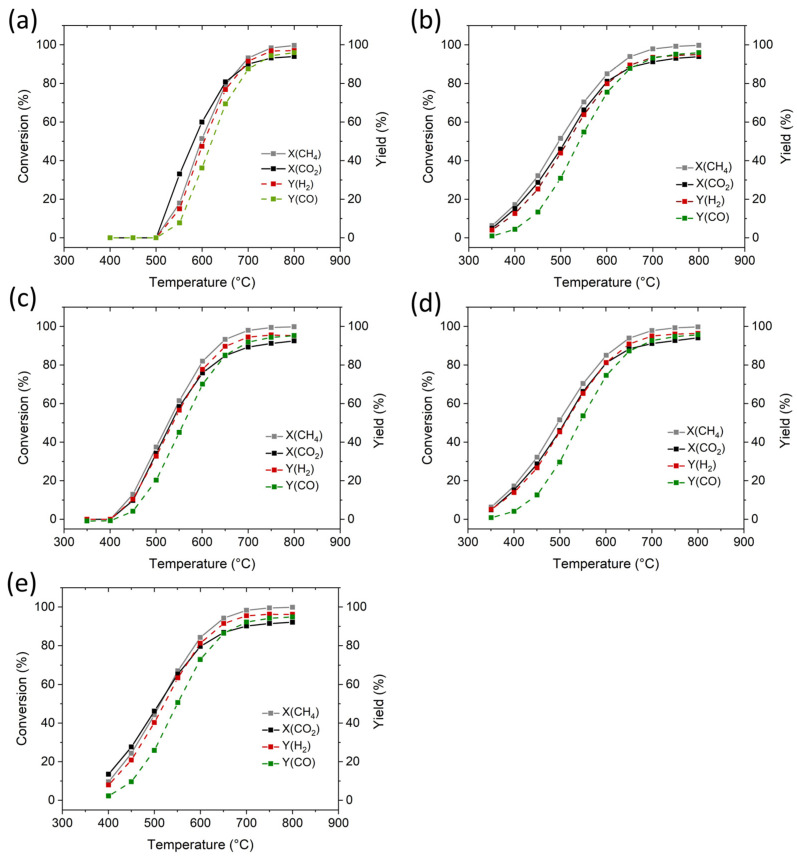
Catalytic performance of reduced aerogel catalysts in DRM test as a function of reaction temperature: (**a**) Sm-Co-O, (**b**) Sm-Ni-O, (**c**) Al-Co-O, (**d**) Al-Ni-O, and (**e**) Al-Co-Ni-O.

The study of catalytic activity and C-deposition in the DRM process catalyzed by monometallic and bimetallic aerogels was used at two time and temperature programs. A mixture of gases CO_2_/CH_4_ = 3.8/3.2 mL/min (1.2/1.0) flowed through the bed of 100 mg of catalyst “gas space velocity” (GHSV) was 36 l/g_cat_**^.^**h. The composition of reactants (CO_2_ and CH_4_) and reaction products (H_2_, CO, and H_2_O) was monitored by MS. The catalytic performance of catalysts during dry reformation of CH_4_ expressed by the conversion of CH_4_ and CO_2_ is shown in Figure 7 and Table 5. From Figure 7, it can be seen that the DRM reaction was initiated on catalysts containing Ni (Sm-Ni-O, Al-Ni-O, and Al-Ni-Co-O) at a temperature of around 350 °C and on catalysts containing Co (Al-Co-O and Sm-Co-O) at a temperature of 400 °C to 500 °C. The conversion of X(CH_4_) increased rapidly when temperature increased from 350 °C to 600 °C and it reached values of up to 100% at 800 °C. The Sm-Co-O catalyst presented lower activity at temperatures below 750 °C due to its shallow surface area (1.51 m^2^/g). The catalytic parameters of the other catalysts were not significantly unaffected by the surface area. There was no significant difference between the values of X(CH_4_), X(CO_2_), Y(H_2_), and Y(CO) of the Al-Ni-O catalyst with a surface area of 68.7 m^2^/g and the Al-Co-O catalyst with a surface area of 11.9 m^2^/g. Instead, the catalytic activity was determined by the density and distribution of the metal particles on the surface, which was similar to these catalysts. The epoxide-driven method, probably at the molecular level (formation of M_i_-O-M_j_ bonds), led to a similar structure of aerogels and reduced catalysts prepared from them. Catalytic tests showed that all aerogel catalysts had excellent activity. The high conversion values of X(CH_4_) and X(CO_2_) catalysts with Ni and Co deposited on the surface of the Al_2_O_3_ substrate (Al-Co-O, Al-Ni-O, and Al-Ni-Co-O) were comparable to those reported in the literature [3,28,29,30,31,32].

**Figure 8:**
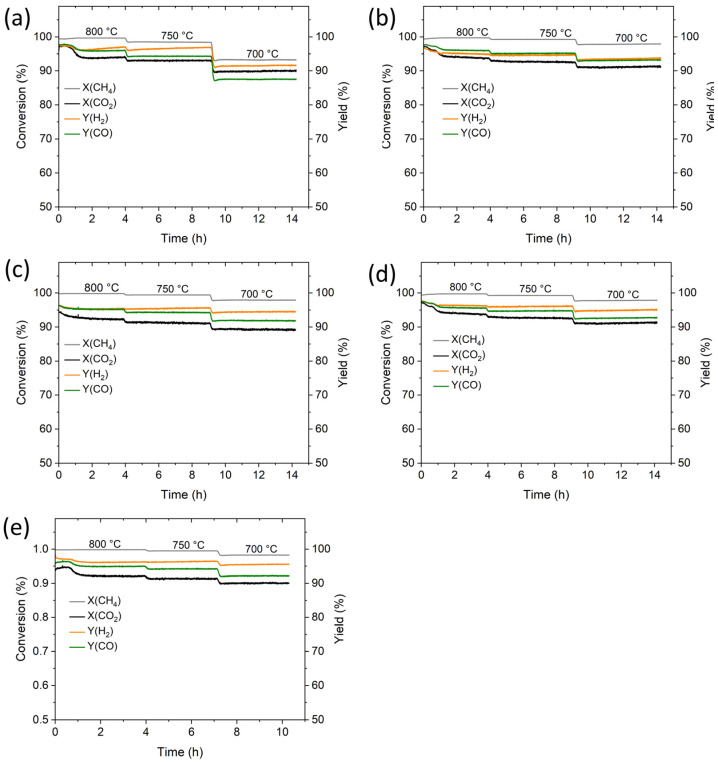
CH_4_ and CO_2_ conversion and H_2_ and CO yield with time on stream in the dry reformation of methane over aerogel catalysts at 800, 750, and 700 °C: (**a**) Sm-Co-O, (**b**) Sm-Ni-O, (**c**) Al-Co-O, (**d**) Al-Ni-O, and (**e**) Al-Co-Ni-O.

The catalytic activity and deactivation of aerogel catalysts can be seen in Figure 8, which shows the time dependence of conversion (X(CH_4_) and X(CO_2_)) and yield (Y(H_2_) and Y(CO)) at three gradually decreasing temperatures (800 °C/4h + 750 °C/5h + 700 °C/5h). CH_4_ and CO_2_ conversions decreased with temperature from 100% to 98% (or 90% in the case of Sm-Co-O) but the time decrease in individual steps was less than 1%. The reason for such minor changes in activity was primarily the short period of DRM testing. The stable behavior of aerogel catalysts under DRM is probably related to the controlled sol–gel synthesis producing M_i_-O-M_j_ mixed bonds.

### 2.4. Characterization of Spent Catalysts

**Figure 9:**
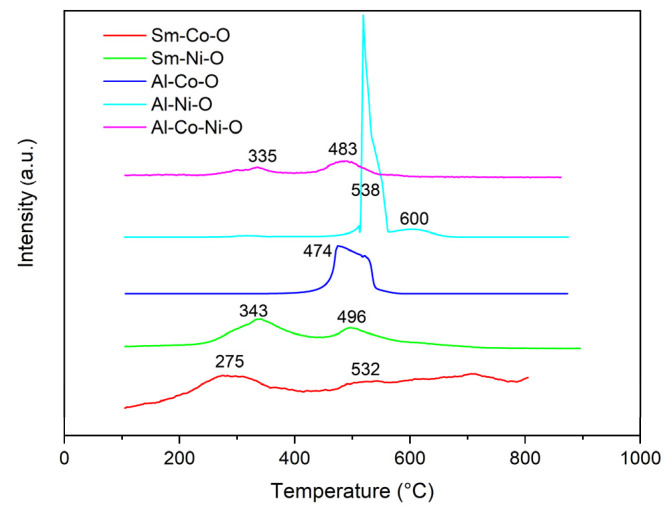
TPO profiles of spent aerogel catalysts (after 14 h of DRM test at 700 to 800 °C).

Temperature-programmed oxidation by oxygen (TPO) profiles of aerogel catalysts after DRM tests (Figure 9) show oxidation peaks at temperatures around 275–335 °C, 474–496 °C and 532–600 °C associated with oxidation of carbonaceous species deposited on the surface catalysts with oxygen. Depending on the temperature, the peaks are assigned to the oxidation of high amorphous, low amorphous, and graphitic carbons [33,34]. The content of the deposit on the surface of the catalyst increased in the order Sm-Co-O < Sm-Ni-O < Al-Co-Ni-O < Al-Ni-O < Al-Co-O (Table 6). C-deposits’ formation depends on the rates of C-deposit formation and its gasification, which is related to the character of the metal and the substrate [35]. The highest C-deposit (2.93 and 4.47 mg C/g_cat_**^.^**h) was found for monometallic metals deposited on Al_2_O_3_ substrates (Al-Ni-O and Al-Co-O). Since the reduction temperature of the high-temperature peak of both aerogels was high (around 900 °C), a strong interaction between Co and Ni and the Al_2_O_3_ substrate can be assumed [36], which should lead to a decrease in the rate of C-deposition on the catalyst surface. Therefore, the cause of the significant C-deposition in these two catalytic systems probably lies in the composition of the metal particles. XPS spectra showed the presence of NiAl alloy in Al-Ni-O and Al-Co-O samples. Due to the presence of metallic Al, a more robust interaction with CO_2_ could occur on the surfaces of NiAl and CoAl alloys, leading to a decrease in the rate of CO_2_ reduction to CO. A higher rate of CH_4_ cracking than CO_2_ reduction led to higher C-deposit production. The very low C-deposition in catalysts containing Sm (Sm-Co-O and Sm-Ni-O) was connected, similarly to La_2_O_3_, to the basic nature of Sm_2_O_3_ [37,38] and the high mobility of oxygen on the active center [37,39]. Electron-deficient Co particles, in combination with a Sm_2_O_3_ carrier, supported the reduction in CO_2_ and the oxidation of C-deposits to CO [27]. The very low C-deposition in the Al-Ni-Co-O catalyst agrees with the published results. The high activity, stability, and low C-deposition of the bimetallic Al-Ni-Co-O catalyst is attributed to the synergistic effect between Co and Ni in the CoNi alloy [40,41]. The synergistic effect resulted in the formation of homogeneously distributed CoNi nanoparticles on the surface of the Al_2_O_3_ substrate and a favorable interaction between CoNi and the substrate. The CH_4_ cracking rate on the CoNi surface was lower than the CO_2_ reduction rate, retarding the C-deposition [42].

**Figure 10:**
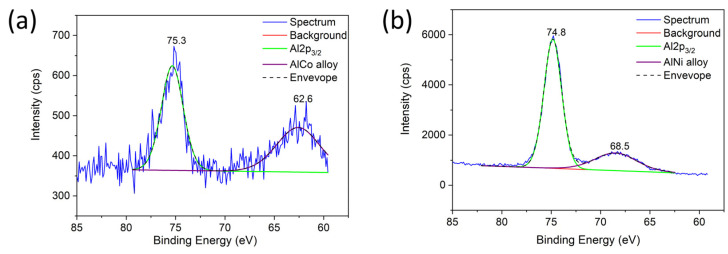
Al 2p XPS spectra of reduced Sm-Al-Co-Ni-O aerogels after DRM tests (**a**) Al-Co-O and (**b**) Al-Ni-O.

XPS analysis of aerogel catalysts after DRM tests confirmed the presence of NiCo alloy in the Al-Co-Ni-O sample based on the difference in binding energy Ni 2p_3/2_ and Co 2p_3/2_ between mono-metallic samples Al-Ni-O, Al-Co-O, and bimetallic Al-Co-Al-O. The difference in Ni 2p_3/2_ binding energies between Al-Ni-O and Al-Co-Al-O was −0.2 eV (857.6 vs. 857.4 eV). The difference between the Co 2p_3/2_ binding energy was +0.45 eV (782.35 vs. 782.8 eV). The migration of Ni 2p_3/2_ peak to higher binding energy and Co 2p_3/2_ peak to higher binding energy indicates electron migration between Ni and Co and confirms the interaction between Co and Ni and the formation of NiCo alloy [43]. Figure 10 shows the XPS spectra of Al-Co-O and Al-Ni-O catalysts after the DRM test containing peaks of 61.8 eV and 68.3 eV assigned to AlCo and AlNi alloys. Due to the difficult reducibility of Al^3+^ with hydrogen, the mentioned alloys were probably only formed during DRM tests at high temperatures (800 °C) by the action of reactive carbon species (C_x_H_y_, CO, and C). In contrast to the “anti-coking” effect of the Al-Ni-Co-O catalyst containing the NiCo alloy, the Al-Co-O and Al-Ni-O catalysts produced the highest amount of C-deposit. AlCo and AlNi alloys containing reactive Al can form a stronger bond with CO_2_, retarded CO release, and oxidation of C-deposit on the catalyst surface.

**Figure 11:**
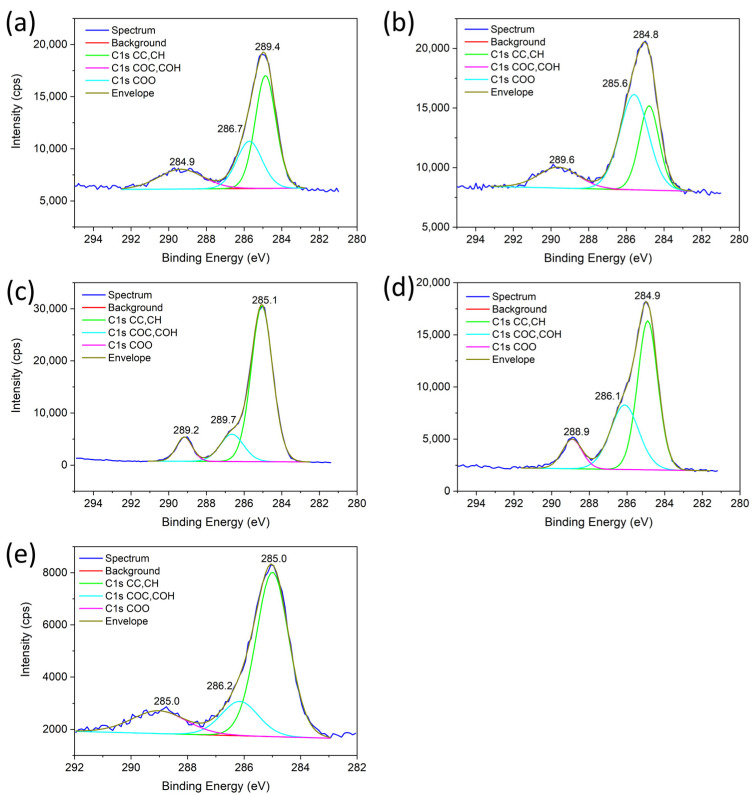
C 1s XPS spectra of aerogels after DRM tests: (**a**) Sm-Co-O, (**b**) Sm-Ni-O, (**c**) Al-Co-O, (**d**) Al-Ni-O, and (**e**) Al-Co-Ni-O.

C(1s) XPS spectra of aerogel catalysts are shown in Figure 11. Deconvoluted peaks centered at the binding energy 284.8–285.1 eV were assigned to C=C and CH groups; peaks at binding energy 285.6–286.7 eV were assigned to functional groups C-OH and C-O-C and peaks 288.8–289.6 eV to CO-O and CO_2_ groups [44,45,46,47]. The relative content of carbon deposit (CC, CH) on Sm-Al-Co-Ni-O catalysts increased in the order Al-Co-O > Al-Ni-Co-O > Al-Ni-O > Sm-Co-O > Sm-Ni-O. The relative content of oxidized C-products (COH, COC, and COO) increased in the opposite order. The highest content (COH, C-O-C, and COO) associated with Sm_2_O_3_ substrates shows that similar to La_2_O_3_, Sm_2_O_3_ participated, due to its basic and redox nature, in the elimination of C_x_H_y_ species from the surface of the catalysts by interaction with CO_2_ [37,38,39].

## 3. Conclusions

Epoxide-driven sol–gel synthesis of multi-cationic aerogels led to dense di- and tri-cationic Sm-Co-O, Sm-Ni-O, Al-Co-O, Al-Ni-O, and Al-Ni-Co-O aerogels. In the course of hydrolysis and condensation (olation and oxolation) of aqua-cationic complexes in the water-ethanolic solution formed not only bonds Sm-O-Co, Sm-O-Ni, Al-O-Co, Al-O-Ni but also M_i_-O-M_i_ bonds which led along the thermal processing to multiphase products. Aerogels heat-treated at a relatively low temperature (500 °C/1 h) had a very high surface area of up to 470 m^2^/g, which decreased more than ten times after calcination at 900 °C/10 h. The aerogels did not have a mesoporous structure and were formed by agglomerates of hydroxide-oxide nanoparticles of Co^2+^, Ni^2+^, Al^3+^ and Sm^3+^. Reduction in di-cationic Sm-Co-O, Sm-Ni-O, Al-Co-O, and Al-Ni-O aerogels resulted in catalysts with heterogeneous distribution of Co^0^ and Ni^0^ nanoparticles and microparticles on the surface of Sm_2_O_3_ and Al_2_O_3_. In addition to tiny, several nanometer-sized nanoparticles uniformly deposited on the surface, spherical particles or clusters of 500–1000 nm size were present. Large particles could form in two-phase Sm-Ni-O, Al-Co-O, and Al-Ni-O catalysts, in which one phase was reduced more easily faster (NiO and Co_3_O_4_) than the other (spinel phase). A uniform distribution of Co^0^ and Ni^0^ nanoparticles (in the form of a CoNi alloy) without large spherical particles was found only in the Al-Co-Ni-O catalyst. Catalytic tests showed that all aerogel catalysts had excellent activity. The epoxide-driven method probably, at the molecular level (formation of M_i_-O-M_j_ bonds), led to a similar structure of aerogels and catalysts prepared from them. The content of C-deposit on the surface of the tested catalysts increased in the order Sm-Co-O < Sm-Ni-O < Al-Co-Ni-O < Al-Ni-O < Al-Co-O. The most C-deposit was found for metals, Co^0^ and Ni^0^, lying on the surface of Al_2_O_3_ substrates (Al-Ni-O and Al-Co-O). Therefore, the cause of the significant C-deposition in these two catalytic systems probably lies in the composition of the metal particles. XPS spectra showed the presence of NiAl alloy in Al-Ni-O and CoAl alloy in Al-Co-O samples. Due to the presence of metallic Al, strong interaction with CO_2_ could occur on the surfaces of NiAl and CoAl alloys, leading to a retarding of CO_2_ reduction. A higher rate of CH_4_ cracking than CO_2_ reduction led to higher C-deposit production. The low C-deposition in catalysts containing Sm (Sm-Co-O and Sm-Ni-O) was connected, similarly to La_2_O_3_, to the basic nature of Sm_2_O_3_ and the high mobility of oxygen on active centers.

## 4. Materials and Methods

### 4.1. Synthesis of Sm-Co-O, Sm-Ni-O, Al-Co-O, Al-Ni-O, and Al-Ni-Co-O Aerogels

Aerogels were prepared using the sol–gel method based on a ring-opening polymerization mechanism. Transition metal chlorides (CoCl_2_**^.^**6H_2_O and NiCl_2_**^.^**6H_2_O) in combination with samarium (SmCl_3_**^.^**6H_2_O) or aluminum chloride (AlCl_3_**^.^**6H_2_O) were used as the starting reactants and were dissolved in ethanol. The molar ratio of the reactants and the concentration of solutions are given in Table 1. Initially, solutions were prepared and stirred for 1 h. Then, propylene oxide was addedand the mixtures were stirred for several minutes and left in the vessels to gel. Gel times are given in Table 1. As a result, jelly samples were aged for 24 h in vessels and then in an acetone environment following supercritical drying, obtaining the aerogels. Aerogels were thermal treated (air, 500 °C/1 h), calcined (air, 900 °C/10 h) and reduced (5 vol.%H_2_ in Ar, 800 °C/1 h) afterward.

### 4.2. Characterization of Aerogels

Aerogels were evaluated via nitrogen adsorption, X-ray diffraction (XRD), X-ray photoelectron spectroscopy (XPS), temperature-programmed reduction by hydrogen (H_2_-TPR), and temperature-programmed oxidation by oxygen (O_2_-TPO). The phase composition of aerogel particles before and after reduction with H_2_ was determined by the RTG diffractometer (Philips X’pert) in a central focusing configuration, using CoKa radiation and two types of detectors, the X’celerator and the Microprobe. The corresponding standards were established by comparing the ICSD (Inorganic Crystal Structure Database, FIZ). The crystallographic structures and quantitative phase-analysis were assessed using the Highscore program (PANalytical). The data were entered into the algorithm according to Rietveld [48] and mathematically processed to cover the measured diffractogram completely. The morphology of the aerogel particles (particle shape and size) and their chemical composition were evaluated on a field-emission scanning electron microscope Verios 460L (Thermo Fisher) equipped with an energy dispersion spectrometer (SEM-EDX). The specific surface of perovskites was measured on a CHEMBET 3000 device (Quantachrome) and, using the BET method, evaluated from the adsorption isotherms of nitrogen measured at 77 K on samples degassed at 300 °C. X-ray photoelectron spectroscopy (XPS) of aerogels reduced in the course of DRM was carried out using Kratos Axis Supra (Kratos-XPS), with monochromatic Ka radiation, 300 W emission power, and magnetic lens and the charge compensation turned on. The survey and detailed spectra were measured using pass energies of 160 and 20 eV, respectively. The spectra were evaluated using the Unifit 2013 software.

H_2_-TPR and O_2_-TPO profiles and simultaneous thermal analysis of aerogels were measured on STA 409 CD/QMS 403 Skimmer analyzers. In total, 20 mg of samples was placed in an Al_2_O_3_ crucible with a reduction or oxidation atmosphere flowing around it. Thermal analysis was measured in a hydrogen or oxygen atmosphere in a temperature range of 50 °C to 950 °C at a heating rate of 10 °C/min. The sample was cooled under an Ar atmosphere at a rate of 10 °C/min. A mixture of 5% H_2_ in Ar and 20% O_2_ in Ar was used, flowing through the thermal analyzer at a rate of 100 mL/min. The mass analysis of the gaseous phase was conducted using the Skimmer device in combination with the thermogravimetric analysis.

### 4.3. Tests of Catalytic Activity of Aerogel Catalysts

Catalytic activity testing of aerogel catalysts was performed on the high-temperature heterogeneous reactor Catlab (Hiden Analytical Ltd., Warrington, UK) from 30 °C to 800 °C at a heating rate of 10 °C/min. The gaseous phase was analyzed using the HPR-20 mass spectrometer (Hiden Analytical Ltd., UK). Temperature-programmed reduction by hydrogen (H_2_-TPR) and temperature-programmed oxidation by oxygen (O_2_-TPO) analyses of each sample were repeated three times. The mass spectrometer was calibrated with two calibration mixtures of gases (CO, CO_2_, H_2_, CH_4_ in Ar, and H_2_O in Ar, Messer, Bridgewater, NJ, USA).

The catalytic bed made up of aerogel particles of 100 mg in weight, deposited on alumina fibers (Saffil ceramic fibers, Unifrax Ltd., Widnes, UK), formed a piston of 40 mm in length. A temperature programmer controlled the furnace temperature in a temperature range of 30 °C to 800 °C. The reaction mixture of model biogas, CH_4_/CO_2_/Ar (3.8/3.2/53 mL/min), flowed through the tubular fixed-bed reactor at an overall rate of 60 mL/min (GHSV = 36 L/g_cat_**^.^**h). The catalytic tests were conducted under isothermal conditions in steps of 100 °C in the range from 400 °C to 1000 °C, with isothermal steps taking 30 min under isobaric conditions at a pressure of 1 bar. Long-time-on-the-stream tests of catalytical stability were conducted at 900 °C/15 h under the conditions given above. Prior to catalytic tests, the aerogels were reduced in Ar/5vol.%H_2_ atmosphere at 800 °C/1 h. Data from the mass spectrometer were used to assess the molar flows of CH_4_, CO_2_, H_2_, and CO. The conversion of methane X(CH_4_), carbon dioxide X(CO_2_), the yield of hydrogen Y(H_2_) and carbon monoxide Y(CO) were calculated according to Equations (5)–(8), where F_i_ is the molar flow rate of i-component at the entrance to the reactor or output from the reactor.

(5) $$X_{CO2}=\frac{F_{CO2(in)}-F_{CO2(out)}}{F_{CO2(in)}}\cdot 100\left[\%\right]$$

(6) $$X_{CH4}=\frac{F_{CH4(in)}-F_{CH4(out)}}{F_{CH4(in)}}\cdot 100\left[\%\right]$$

(7) $$Y_{H_{2}}=\frac{F_{H_{2(out)}}}{2F_{{CH}_{4(in)}}}\cdot 100\left[\%\right]$$

(8) $$Y_{CO}=\frac{F_{CO(out)}}{F_{CH4(in)}+F_{CO_{2(in)}}}\cdot 100\left[\%\right]$$

## Figures and Tables

**Table 1:** Summary of synthesis conditions in the preparation of Sm-Co-O, Sm-Ni-O, Al-Co-O, Al-Ni-O, and Al-Co-Ni-O gels.

Synthesis Precursors	Concentration (mol)	H_2_O/M_i_ (mol/mol)	t_gel_ (min)	Charge Density of Cations (C/mm^3^)
CoCl_2_·6H_2_O	0.0144	6	25	155
SmCl_3_·6H_2_O	0.0144			86
propylene oxide	0.2858			
ethanol	1.182			
NiCl_2_·6H_2_O	0.0144	6	7	134
SmCl_3_·6H_2_O	0.0144			86
propylene oxide	0.2858			
ethanol	1.182			
CoCl_2_·6H_2_O	0.0144	6	3	155
AlCl_3_·6H_2_O	0.0144			364
propylene oxide	0.2858			
ethanol	1.182			
NiCl_2_·6H_2_O	0.0144	6	3	134
AlCl_3_·6H_2_O	0.0144			364
propylene oxide	0.2858			
ethanol	1.182			
NiCl_2_·6H_2_O	0.0072	6	4	134
CoCl_2_·6H_2_O	0.0072			155
AlCl_3_·6H_2_O	0.0144			364
propylene oxide	0.2858			
ethanol	1.182			

**Table 2:** Simultaneous TGA/MS of supercritically dried aerogels.

Sample/Peak	(Dm)_TG_	(Q)_DSC_	(T_max_)_DTG_	(T_max_)_DSC_	(T_max_)_H2O_	(T_max_)_CO2_	(T_max_)_HCl_
	(wt%)	(J/g)	(°C)				
SmCoO/1	−6	−122	134	120	123		
SmCoO/2	−17	642	287	294	288	288	254
SmNiO/1	−9	−241	144	135	138	134	
SmNiO/2	−7	588	259	260	259	259	226
SmNiO/3	−16		324	324	317	317	
AlCoO/1	−16	−202	134	134	144		
AlCoO/2	−10	363	287	310	286	301	225
AlNiO/1	−12	−162	134	125	140	122	
AlNiO/2	−15	77	291	305	273	293	
AlNiO/3	−5	80	615	376		359	
AlNiCoO/1		−254	136	136	144	136	
AlNiCoO/2		242	289	337	289	330	

**Table 3:** The specific surface of thermally treated and calcined aerogels.

Sample	Specific Surface (m^2^/g)	
	Thermally Treated	Calcined
Sm-Co-O	325	1.51
Sm-Ni-O	470	18.9
Al-Co-O	459	11.9
Al-Ni-O	395	68.7
Al-Co-Ni-O	346	34.4

**Table 4:** Elemental composition of the reduced aerogels obtained from scanning electron microscopy-energy dispersive X-ray spectroscopy (SEM-EDX) analysis.

Sample	At. %				
	Sm	Al	Co	Ni	O
Sm-Co-O	27.7	0.0	25.0	0.0	47.4
Sm-Ni-O	29.0	0.0	24.5	0.0	46.5
Al-Co-O	0.0	28.6	23.4	0.0	48.0
Al-Ni-O	0.0	27.9	0.0	24.8	47.4
Al-Co-Ni-O	0.0	24.2	9.3	25.3	41.2

**Table 5:** Catalytic performance of Sm-Al-Co-N i-O catalysts in dry reformation of methane at 600 to 800 °C.

Sample	Conversion (%)	Temperature (°C)				
	Yield (%)	600	650	700	750	800
Sm-Co-O	X(CH_4_)	51.1 ± 0.3	81.2 ± 0.3	91.8 ± 0.4	98.0 ± 0.4	99.6 ± 0.2
	X(CO_2_)	60.2 ± 0.2	80.4 ± 0.2	90.2 ± 0.3	92.5 ±0.6	93.7 ± 0.5
	Y(H_2_)	46.6 ± 0.2	77.3 ± 0.1	90.6 ± 0.3	96.4 ± 0.4	95.8 ± 0.3
	Y(CO)	36.4 ± 0.1	69.5 ± 0.3	87.4 ± 0.4	93.8 ± 0.3	95.7 ± 0.6
Sm-Ni-O	X(CH_4_)	85.2 ± 0.1	93.8 ± 0.1	97.6 ± 0.1	99.3 ± 0.2	99.8 ± 0.1
	X(CO_2_)	80.6 ± 0.4	87.6 ± 0.3	91.4 ± 0.2	92.5 ± 0.3	94.1 ± 0.3
	Y(H_2_)	88.4 ± 0.2	88.7 ± 0.2	93.2 ± 0.2	93.7 ± 0.2	94.6 ± 0.4
	Y(CO)	76.3 ± 0.1	80.1 ± 0.2	92.7 ± 0.3	92.6 ± 0.2	96.3 ± 0.1
Al-Co-O	X(CH_4_)	82.2 ± 0.1	92.6 ± 0.5	97.6 ± 0.2	99.3 ± 0.4	99.7 ± 0.2
	X(CO_2_)	76.4 ± 0.2	84.0 ± 0.3	89.2 ± 0.1	91.2 ± 0.2	92.3 ± 0.5
	Y(H_2_)	76.6 ± 0.2	88.6 ± 0.1	94.3 ± 0.2	94.7 ± 0.2	95.4 ± 0.1
	Y(CO)	70.4 ± 0.1	85.2 ± 0.6	92.4 ± 0.1	94.6 ± 0.2	95.2 ± 0.2
Al-Ni-O	X(CH_4_)	85.1 ± 0.3	93.7 ± 0.2	98.4 ± 0.1	99.2 ± 0.2	99.9 ± 0.1
	X(CO_2_)	80.6 ± 0.1	88.4 ± 0.2	91.3 ± 0.4	93.1 ± 0.2	94.6 ± 0.2
	Y(H_2_)	80.5 ± 0.4	91.0 ± 0.3	94.7 ± 0.1	95.6 ± 0.5	96.4 ± 0.2
	Y(CO)	74.6 ± 0.2	87.4 ± 0.2	93.3 ± 0.2	94.5 ± 0.3	95.7 ± 0.2
Al-Co-Ni-O	X(CH_4_)	84.3 ± 0.2	84.3 ± 0.1	97.5 ± 0.2	99.4 ± 0.2	99.7 ± 0.3
	X(CO_2_)	79.4 ± 0.2	86.5 ± 0.2	89.6 ± 0.2	91.3 ± 0.4	92.3 ± 0.2
	Y(H_2_)	81.1 ± 0.1	92.3 ± 0.4	95.3 ± 0.3	96.4 ± 0.3	95.7 ± 0.2
	Y(CO)	73.1 ± 0.3	86.2 ± 0.2	92.1 ± 0.2	94.0 ± 0.6	95.3 ± 0.1

**Table 6:** Carbon deposition after 14 h of methane dry reformation catalyzed by aerogel catalysts.

Sample/Peak	T_CO2_ (°C)		IC_CO2_ (A·s·10^9^)		C-Deposit (mg C/gcat^.^h)		SC-Deposit	
	Mean	Sdev	Mean	Sdev	Mean	Sdev	Mean	Sdev
Sm-Co-O/1	275	3	3.8	0.6	0.028	0.011	0.042	0.021
Sm-Co-O/2	532	6	1.9	0.3	0.014	0.01		
Sm-Ni-O/1	343	22	35.3	5.2	0.26	0.005	0.29	0.008
Sm-Ni-O/2	496	5	4.3	0.6	0.03	0.004		
Al-Co-O/1	484	16	607.7	89.4	4.47	0.43	4.47	0.43
Al-Ni-O/1	538	3	282.7	41.6	2.08	0.023	2.93	0.077
Al-Ni-O/2	600	3	115.3	17.0	0.85	0.074		
Al-Co-Ni-O/1	335	5	14.4	2.1	0.11	0.005	0.35	0.011
Al-Co-Ni-O/2	483	4	33.1	4.9	0.24	0.006		

## Data Availability

The original contributions presented in the study are included in the article, further inquiries can be directed to the corresponding author.
